# Conservative Management of Ovarian Fibroma in A Case of Gorlin-Goltz Syndrome Comorbid with Endometriosis 

**DOI:** 10.22074/ijfs.2018.5240

**Published:** 2018-01-15

**Authors:** Sepideh Khodaverdi, Leila Nazari, Abolfazl Mehdizadeh-Kashi, Mansoureh Vahdat, Samaneh Rokhgireh, Ali Farbod, Banafsheh Tajbakhsh

**Affiliations:** 1 Endometriosis Research Center, Iran University of Medical Science, Tehran, Iran; 2 Department of General Surgery, Rasool-Akram Hospital, Iran University of Medical Science, Tehran, Iran

**Keywords:** Endometriosis, Gorlin-Goltz Syndrome, Odontogenic Keratocysts, Ovarian Fibroma

## Abstract

Ovarian fibromas are the most common benign solid ovarian tumors, which are often difficult to diagnose preoperatively. Ovarian fibromas, especially in bilateral cases, may be cases of Gorlin-Goltz syndrome (GGS), a rare autosomal dominant disorder with predisposition to basal cell carcinomas (BCCs) and other various benign and malignant tumors. This case report describes a 25 year-old female with GGS, bilateral ovarian fibroma, endometriosis and septated uterus, which was referred to the Gynecology Clinic of Rasoul-e-Akram Hospital in October 2016. This patient had facial asymmetry due to recurrent odontogenic keratocysts. In young cases of ovarian fibromas as reported here, conservative surgical management can preserve ovarian function and fertility. These patients must be followed up by a multidisciplinary team and submitted to periodic tests.

## Introduction

Gorlin-Goltz syndrome (GGS), also known as the nevoid basal cell carcinoma syndrome (NBCCS), is an autosomal dominant inherited disorder (1). The incidence of this disorder is estimated to be 1 in 50,000 to 150,000 in the general population, varying by geographic region (2). Although it occurs in all ethnic groups, it mostly affects whites, with males and females equally affected (3). Pathogenesis of NBCCS is due to mutations in the patched tumor suppressor gene 9q22.32; PTCH1 causing abnormality in the Hedgehog (Hh) signaling pathway, thus resulting in neoplasm formation (4).

GGS is characterized mainly by the presence of multiple basal cell carcinomas (BCC), odontogenic keratocysts (OKCs) of the jaw, palmar pits and ectopic calcifications of the cerebral falx. More than a 100 minor criteria have also been described. The presence of two major and one minor criteria or one major and three minor criteria are necessary to establish a diagnosis (5). Recent consensus statement from the first international colloquium on basal cell nevus syndrome(BCNS) proposed less stringent criteria for diagnosis where one major criterion and molecular confirmation, two major criteria or one major and two minor criteria are sufficient (Table 1) (3).

**Table 1:** Criteria for diagnosis Gorlin-Goltz syndrome


The major criteria are:	The minor criteria are:

Multiple BCC or one occurring under the age of 20 years	Macrocephaly (adjusted for height)
Histologically proven OKCs of the jaws	Congenital malformation: cleft lip/palate, frontal bossing, coarse face, moderate or severe hypertelorism
Palmar or plantar pits (three or more)	Other skeletal abnormalities: sprengel deformity, marked pectus deformity, marked syndactyly of the digits
Bilamellar calcification of the falx cerebri	Radiological abnormalities: bridging of the sella turcica, vertebral anomalies such as hemivertebrae, fusion or elongation of the vertebral bodies, modeling defects of the hands and feet or flame shaped hands or feet
Bifid, fused or markedly splayed ribs	Ovarian fibroma
A first-degree relative with NBCCS	Medulloblastoma


BCC; Basal cell carcinoma, OKCs; Odontogenic keratocysts and NBCCS; Nevoid basal cell carcinoma syndrome

Early diagnosis of the syndrome is of great clinical importance since the severity of complications, such as maxillofacial deformities related to the jaw cyst, can be avoided and long-term prognosis of malignant skin lesion and brain tumor is better when early diagnosis and treatment is initiated (6).

Diagnosis of NBCCS may be difficult because of variable expressivity and different age-onsets for different traits of this disorder. The average age for diagnosis of NBCCS is 13 years while the average age for detection of basal cell carcinoma is 20 years. The clinical expression of the syndrome varies among individuals within the same family and to a greater extent among families (7). This case report describes a patient with typical features of GS, diagnosed for the first time in our Department.

## Case report

A 25 year-old female was referred to the Gynecology Clinic of Rasoul-e-Akram Hospital because of chronic abdominal pain, myomatous uterus and a 6×8 cm^2^ right adnexal mass suspicious to be a dermoid cyst in sonography and magnetic resonance imaging (MRI) reports due to the presence of dense calcification in the tumor. Tumor markers were all normal. She had been born by uncomplicated normal vaginal delivery. She spoke and walked at 19 months of age and her neurodevelopment was normal. At 21 years of age, she was diagnosed with OKCs in the mandibular and maxillary regions, and submitted to surgery for the removal of her dental cysts. In less than a year, the surgery was repeated due to recurrent OKCs.

One year later, she complained of pain in her lower abdomen and underwent trans-abdominal ultrasonography and pelvic MRI, which revealed the right ovarian mass, suspicious of being a dermoid cyst, and a myomatosis septated uterus. Physical examination revealed hirsutism with harsh face and multiple nevi on face and upper trunk. She underwent laparoscopic surgery but after abdominal entry, we encountered unusual round solid ovarian masses, which could not be excluded as malignant. Frozen sections, however, showed they were benign. There was a 6 cm endometrioma in the right ovary and multiple bilateral ovarian fibromas (Figes.1, 2). There were also endometriotic patches in a posterior cul-de-sac. We excised 7 fibromas from the left ovary and 5 fibromas from the right ovary in different sizes ranging from 0.3 to 5 cm in diameter. Both ovaries were preserved. The definitive histologic diagnosis confirmed stromal proliferation and no atypia in the ovarian tissue with areas of necrosis, corresponding to an ovarian fibroma and endometriosis.

According to the laparoscopy outcome, pathologic findings and history of recurrent OKCs, Gorlin syndrome was the top differential diagnosis. Investigation for other signs and symptoms of this syndrome confirmed the diagnosis. Chest radiography, posterior-anterior skull view and spine x- rays were normal. We referred the patient to a dermatologist and excisional biopsy of nevi was undertaken. Fortunately, the pathologic examination was benign. A written consent was taken from the patient for publication of this report.

**Fig.1:**
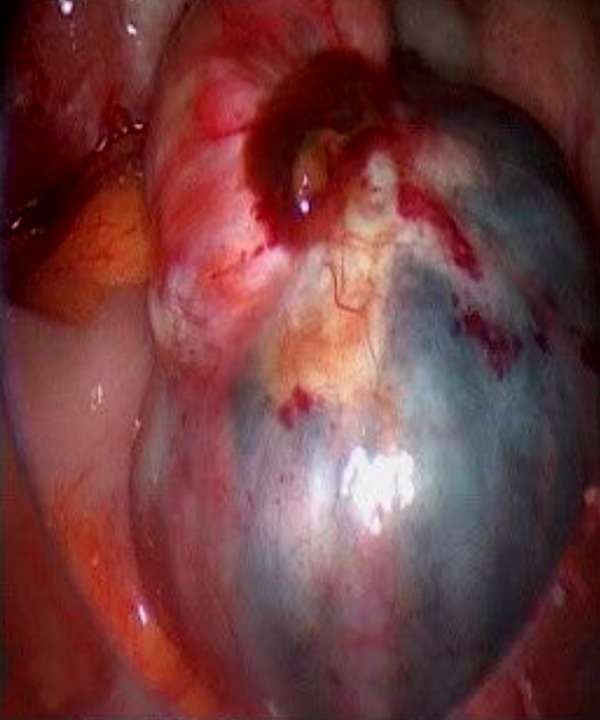
The right ovary with endometrioma and fibroma.

**Fig.2:**
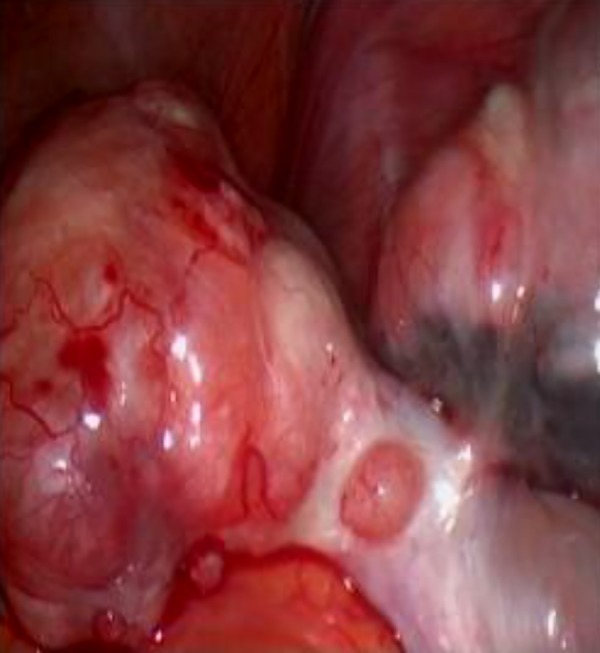
The left ovary with endometrioma and fibroma.

## Discussion

GS is an autosomal dominant disorder with near complete penetrance and variable expressivity, and with an estimated birth incidence of 1 in 19,000 individuals (2).

Our patient had one major criterion (i.e. multiple OKCs in the jaw) and 2 minor features (i.e. multiple bilateral ovarian fibromas and a coarse face), thus suggesting it to be a case of GGS.

## Conclusion

Three quarters of female patients with GGS are affected with ovarian fibroma that could be bilateral and recurrent, and thus requires repeated surgery. Fertility of the patient may be influenced by these repeated surgeries and one of the important consultations with these patients has to be about fertility preservation plans. Ovarian fibromas can be excised with minimally invasive methods and the function of the ovary can be preserved at a healthier state.
